# Transmitted Drug Resistance among People Living with HIV/Aids at Major Cities of Sao Paulo State, Brazil

**DOI:** 10.1155/2013/878237

**Published:** 2013-01-15

**Authors:** Joao Leandro Paula Ferreira, Rosangela Rodrigues, Andre Minhoto Lança, Valeria Correia de Almeida, Simone Queiroz Rocha, Taisa Grotta Ragazzo, Denise Lotufo Estevam, Luis Fernando de Macedo Brigido

**Affiliations:** ^1^Laboratório de Retrovírus, Centro de Virologia, Instituto Adolfo Lutz, Avenue Dr. Arnaldo 355, 01246-902 São Paulo, SP, Brazil; ^2^Centro de Referência em DST/Aids, 13013-051 Campinas, SP, Brazil; ^3^Centro de Referência e Treinamento em DST/Aids, 04121-000 São Paulo, SP, Brazil

## Abstract

Human immunodeficiency virus type 1 (HIV-1) transmitted drug resistance (TDR) is an important public health issue. In Brazil, low to intermediate resistance levels have been described. We assessed 225 HIV-1 infected, antiretroviral naïve individuals, from HIV Reference Centers at two major metropolitan areas of Sao Paulo (Sao Paulo and Campinas), the state that concentrates most of the Brazilian Aids cases. TDR was analyzed by Stanford Calibrated Population Resistance criteria (CPR), and mutations were observed in 17 individuals (7.6%, 95% CI: 4.5%–11.9%). Seventy-six percent of genomes (13/17) with TDR carried a nonnucleoside reverse transcriptase inhibitor (NNRTI) resistance mutation, mostly K103N/S (9/13, 69%), potentially compromising the preferential first-line therapy suggested by the Brazilian HIV Treatment Guideline that recommends efavirenz-based combinations. Moreover, 6/17 (35%) had multiple mutations associated with resistance to one or more classes. HIV-1 B was the prevalent subtype (80%); other subtypes include HIV-1 F and C, mosaics BC, BF, and single cases of subtype A1 and CRF02_AG. The HIV Reference Center of Campinas presented more cases with TDR, with a significant association of TDR with clade B infection (*P* < 0.05).

## 1. Introduction

Access to free antiretroviral therapy (ART) is part of the Brazilian response to the Aids epidemic and transmitted drug resistance (TDR); it has been a concern since the introduction of highly active antiretroviral therapy (HAART) in the late 1990s [1]. TDR surveillance is an important strategy to monitor the emergence of genetic resistance as it may impact ART efficacy [2]. This issue was especially sensible in Brazil that deployed a free ARV program in the late 90s amidst a suboptimal health care system. This initiative could boost the emergence of transmitted drug resistance variants and jeopardize Human immunodeficiency virus (HIV-1) treatment [3]. However, most studies in Brazil have shown TDR prevalence similar to that observed among developed countries. Two recent Brazilian national surveys had accessed this issue [4, 5] but included a small representation of São Paulo metropolitan areas. We and others have analyzed mutations in treatment-naive individuals [6–12]; but to trace trends for TDR prevalence, continual monitoring is necessary. 

We analyzed ARV naïve individuals living with HIV/Aids, recruited at HIV Reference Centers from the two major metropolitan areas of Sao Paulo state to investigate the TDR prevalence. These metropolitan areas concentrate 44% of notified Aids cases of Sao Paulo state and about one third of the Brazilians living with HIV/Aids.

## 2. Materials and Methods

People living with HIV, asymptomatic and naïve to ART were recruited at outpatient Clinic or Voluntary Counseling and Testing (VCT) at two metropolitan areas, the HIV State Reference Center at the city of Sao Paulo and the Municipal HIV Reference Center at the city of Campinas. Volunteers' selection was conducted by their primary physicians' or by VCT counseling personnel, with additional revision to confirm ARV exposure history. Individuals that agreed to participate were interviewed by clinical staff to access risk, review of potential previous exposures to ART (e.g., MTCT or postexposure prophylaxis), and document the knowledge of a partnership (sexual or sharing of drug paraphernalia) with individuals using ARV. Blood samples were collected from May 2008 to November 2009. Briefly, HIV-1 RNA was extracted with QIAamp viral RNA mini kit (Qiagen, Germany) and reverse transcribed with random primers and Superscript III enzyme (Invitrogen, USA). In samples of low HIV-1 RNA viral load or negative plasma detection, DNA from peripheral blood mononuclear cells (PBMCs) was extracted (Qiagen, Germany). Nested PCR products were sequenced using Big Dye terminators at an ABI 3100 Genetic Analyzer (ABI, USA) to evaluate protease (PR, codons 1 to 99) and partial reverse transcriptase (RT, codons 1 to 235) genes as previously described [6]. Sequences were manually edited using Sequencher 4.7 software (Gene Codes, USA). Ambiguous DNA bases (mixtures) at resistance associated codons were considered at sequence edition. HIV genotyping resistance test results were reported to the HIV Reference Centers. TDR was defined according to the Calibrated Population Resistance Version 6.0 (CPR, Stanford Database, SDRM 2009), an algorithm specifically designed for the epidemiologic surveillance of HIV-1 transmitted drug resistance mutations (DRMs) [13]. International Antiviral Society (IAS) 2011 resistance list [14] was additionally considered to evaluate the impact of resistance in ART response, which considers all mutations that impact ARV susceptibility. To contribute to HIV molecular epidemiology surveillance, HIV-1 subtyping was performed at NCBI genotyping and REGA HIV subtyping tools and confirmed by phylogenetic methods (PAUP* 4.10b), using evolution model selected by ModelTest3.7. Sequences are available at GenBank with accession numbers: HM533970 to HM534205; HQ015155 to HQ015157.

The Institutional Review Boards of the participating HIV Reference Centers and Adolfo Lutz Institute approved this study. 

## 3. Results

Of the 243 HIV-1 infected individuals enrolled in the study, partial HIV-1 *pol* sequences from 230 (95%) individuals were successfully sequenced (96% from plasma and 4% from PBMC). The inclusion criterion of no previous exposure to ART was met by 225 individuals and included in the analysis. Among the study individuals, six females had previous exposure to MTCT prophylaxis, documented at the interview. Most of the sequences analyzed from these women (3/4) exhibited one or more DRM but were not included in TDR prevalence estimate that was generated from the 225 ART naive individuals. Also, one case with unknown information of exposure to ART, but with history of undetectable viremia in previous years, had several resistance mutations and was excluded from analysis. Unprotected exposure among men who have sex with men (MSM) was the most frequent transmission route.  Table 1 depicts demographic and laboratorial data from study volunteers at Campinas and São Paulo sites. 

According to Stanford CPR, TDR mutations were detected in 17 sequences (7.6%, 95% CI: 4.5%–11.9%). The prevalence of TDR at Campinas was 9.6% (15/156) and at Sao Paulo 2.9% was (2/69) (*P* = 0.13). The two individuals from Sao Paulo site had more extensive TDR patterns, both with mutations to protease inhibitor (PI) and NNRTI and one with additional resistance mutations to nucleoside reverse transcriptase inhibitor (NRTI) (Table 2). At Campinas site, most TDR were NNRTI-resistance mutations (73%, 11/15), followed by PI and NRTI (20%, 3/15 each), and 2 sequences were resistant to two ARV classes. Overall, 76% (13/17) of sequences bearing at least one TDR had an NNRTI resistance, 71% (12/17) impairing susceptibility to efavirenz (EFV). K103N/S (53%, 9/17) was the most frequent mutation observed. No association of TDR to transmission risk group or gender was observed. Also, individuals referring sexual partner on ART have a similar number of TDR (*P* = 0.8). Campinas tended to have more cases with TDR, with a significant association of TDR to HIV-1 B infection using either CPR or IAS list (*P* < 0.05).

To evaluate the impact of mutations or polymorphisms associated to resistance, we additionally used the 2011 IAS updated mutation list, and 32 sequences (14.2%) had at least one IAS major mutation (Table 2). Both IAS and CPR criteria were used to assess the relevance of mutations on successful therapy in patients where, according the Brazilian treatment guidelines, ART was recommended. The genotyping test reports were available to the HIV Reference Centers, and the ART was selected taking into account the test report. Virological responses of patients with TDR were assessed until 2012 (Table 2). Out of the 33 individuals with drug resistance mutation, 18 initiated ART, and 16 had clinical and laboratory information, some cases with followup of 37 months. This followup showed that all but one individual (BR09CA175), who had documented adherence issues, were virally suppressed at last observation (Table 2). 

## 4. Discussion

Among this population of antiretroviral naïve HIV-1-infected individuals attending the Campinas and São Paulo Reference Centers, transmitted drug resistance was detected overall in 7.6% of individuals. This TDR prevalence was similar to that reported in other regions of Brazil that used Stanford CPR criteria for these estimates, [4–12, 15]. The reasons for this stabilization, or even decrease of TDR prevalence in most surveys are unclear, but there may well be a plateau where the circulation of mutated isolates may come to some equilibrium, depending on multiple factors as the ARV therapy usage, therapy combinations, adherence, and social networking, among others. The followup of the small but consistent increase in TDR in Africa after the introduction of treatment programs [2] will allow verifying this hypothesis. 

In this study, most individuals had high CD4+ T cell counts at collection, with a median of 463 cells/mm^3^. However, 25% had CD4+ T cell counts below 350 cells/mm^3^, CD4 counts that were indicative of ARV treatment by Brazilian Guidelines at the time. Currently, the recommendation is starting therapy when CD4+ T cells counts drops below 500 cells/mm^3^. Although not the focus of this study, a small number of women exposed to MTCT prevention were recruited but excluded in TDR estimates. These women had significant resistance profile and constitute a population segment that should have access to pretreatment genotype test. 

In Brazil, over 200,000 patients are currently using ART, with 80,000 in São Paulo state. However, some of these patients are not virally suppressed, representing a potential source of transmitted drug resistance. On the other hand, a large number of untreated individuals harbor wild-type variants (estimated to be at least twice the number of treated individuals), another source of HIV transmission that may play an important role in the low prevalence of documented transmitted resistance, consistent across most studies. Additionally, low fitness or other biological limitations in the transmissibility potential of these variants might also have a role in the observed prevalence of resistant strains. More important than point estimates of transmitted resistance would be to trace its tendency in time. Trends in TDR based on sequential assess of sentinel sites are probably one of the best ways to monitor TDR. The Brazilian AIDS Program have conducted a large TDR survey [4], but differences in methodology, design, and participating sites at a previous study [8] limit the comparability of these evaluations. Other recent national study [5], conducted almost at the same time, indicate similar numbers, with estimates at 5–15%, applying the HIV Threshold Survey methodology from WHO. For sites in the Sao Paulo state, the estimates generated at these studies ranged from 0 to 15% [4, 5]. However, these studies had small samples size at sites in the state, ranging from 7 to 34 individuals, which decreased the strength of these to estimates. These differences probably also reflect issues as sampling effects, study design, stringency in the confirmation of ART naïve status, and criteria for defining TDR. Additionally, one of these studies [4] found an association of partnership of individuals using ART with the presence of TDR. This issue was also evaluated here, and the number of TDR was similar among cases reporting a partner using ART. 

As expected for the subtype prevalence at this region of Brazil, HIV-1 subtype B was the most common in both sites. However, the proportions of HIV-1 C and BC mosaics were higher than previously reported in the area. This is further detailed elsewhere [16]. Also, a single CRF02_AG was identified, and although it is the most disseminated HIV recombinant form worldwide, it is rather uncommon at South America, with a report of its presence in Brazil [17]. In this study, we found an association within Campinas sequences of transmitted resistance to subtype B (*P* = 0.05) using either CPR or IAS criteria, in agreement with Sprinz et al. [4], where TDR was predominantly found among subtype B infected individuals.

The impact on treatment efficacy is the central problem of TDR. The high prevalence of NNRTIs resistance among cases with TDR is a potential problem as the Brazilian HIV Treatment Guideline recommends NNRTI, especially efavirenz-based HAART, as a preferential first-line therapy. Considering the low genetic barrier of NNRTI, which a single DRM may confer high level of resistance to the entire class, these patients may actually be initiating ARV with a functional dual therapy, leading to a partial viral suppression, a favoring environment for the emergence of further resistance mutations. Moreover, no decrease in viral fitness is expected for mutations to this class, and they tend to persist longer and be more readily detectable than others that interfere with viral fitness, such as M184V. Persistence as a detectable mutation in population sequencing does not mean the same as persistence throughout the viral quasispecies, and it is conceivable that mutations detected in population sequencing may be considered “sentinel mutations.” These could indicate the presence of additional resistance mutations, below detection limits of this sequencing approach, which could interfere with treatment success [18]. If those detectable mutations signs that other occult mutations exist, one would expect treatment compromise even if physicians have access to pretreatment genotype. Although the number of cases is small, our study documented relatively long-term treatment outcome. As the physicians received the genotype test results, cases initiating HAART after the test result had a therapy influenced by the genotyping test. In most cases a combination therapy was chosen to include drugs not predicted to be affected by the observed DRM. This would have circumvented the direct effect of the detected mutation but not of the occult, additional resistances supposedly present in minority variants. However, the presence of TDR in these individuals did not have an impact in viremia control, and the only treated case without suppression had documented adherence issues.

## 5. Conclusion

We observed low to intermediate levels of transmitted drug resistance mutations (7.6%), with most of them impairing susceptibility to efavirenz, a preferential ARV in first-line therapy at Brazil, confirming previous findings in the country. It is important both to monitor transmitted resistance trends and to define algorithms that might subsidize treatment alternatives where access to genotype test prior to therapy initiation to all individuals may be unrealistic. On the other hand, targeting genotypic test to population segments most susceptible to TDR may be cost effective. This would have both the potential benefit in HIV treatment response and an impact in reducing the drug resistance transmission of to the overall population.

## Figures and Tables

**Table 1:** Demographic and laboratorial characteristics of the study individuals according to transmitted drug resistance (TDR).

	TDR (*n* = 33)	Without TDR (*n* = 192)	Total (*n* = 225)
Age (year old)	32 (29–40)	34 (29–40)	34 (29–40)
Gender (male)	23 (69.7%)	138 (71.9%)	161 (71.6%)
CD4+ T cells (cells/mm^3^)	461 (322–631)	475 (353–623)	463 (344–626)
Viral load (Log_10_)	4.1 (3.6–4.8)	4.3 (3.6–4.7)	4.3 (3.6–4.7)
HIV exposure			
MSM	20 (61%)	100 (51%)	120 (52%)
Heterosexual	11 (33%)	76 (41%)	87 (40%)
IDU	1 (33%)	1 (0.5%)	2 (0.4%)
HIV-1 subtype (partial *pol*)			
A1	0	1 (0.5%)	1 (0.4%)
B	31 (94%)	149 (78%)	180 (76%)
F	0	14 (7%)	14 (6%)
C	1 (33%)	15 (8%)	16 (7%)
CRF02_AG	0	1 (0.5%)	1 (0.4%)
Recombinant mosaic	1 (33%)	12 (6%)	13 (6%)
Surveillance site			
Campinas	26 (79%)	130 (68%)	156 (69%)
São Paulo	7 (21%)	62 (32%)	69 (31%)

**Table 2:** Followup information from individuals with *TDR* associated with ARV resistance.

ID^a^	HIV exposure^b^	Age^c^/gender^d^	ART regimen^e^	CD4+ T cells		Viral load		Time to last VL/CD4 test^j^	Resistance mutations (CPR)^k^	Resistance mutations (IAS)^l^	Subtype^m^
				(cells/mm^3^)		(Log_10_)					
				Before ART^f^	Last test^g^	Before ART^h^	Last test^i^				
BR08SP441	MSM	25/M	TDF/3TC/EFV	*312 *	**311**	*4.40 *	4.78	N.A		E138A	B
BR09SP005	MSM	41/M	AZT/3TC/LPV	388	**1025**	4.77	**<1.70**	13 months		E138A	B
BR09CA065	MSM	37/M	TDF/3TC/EFV	248	**641**	4.43	**<1.70**	8 months		V82I	B
BR09CA067	MSM	38/M	AZT/3TC/EFV	244	**817**	4.58	**<1.70**	31 months		V82I	B
BR09CA071	MSM	29/M	—	*487 *	562	*3.58 *	2.61	—	K103N	K103N	B
BR09CA075	WSM	66/F	AZT/3TC/EFV	224	**378**	5.70	**<1.70**	18 months		E138A	B
BR09CA091	MSM	30/M	TDF/3TC/LPV/r	260	**384**	4.94	**<1.70**	26 months	K103N	K103N	B
BR09CA097	WSM	40/F	—	*567 *	567	*4.18 *	4.18	—	G190A	G190A	B
BR09CA175	MSM	29/M	AZT/3TC/ATV/r	283	164	3.76	3.58	No supression	K103N	K103N	B
BR09CA190	WSM	32/F	AZT/3TC/EFV	207	**609**	4.47	**<1.70**	37 months		E138A	B
BR09CA192	MSM	29/M	—	*751 *	686	*3.74 *	4.25	—	K103N	K103N	B
BR09CA194	IDU+MSM	34/M	N.A.	*230 *	230	—	**<1.70**	N.A.		Q58E	B
BR09CA210	MSM	22/M	—	*741 *	636	*5.19 *	4.90	—	M41L, T215E	M41L	B
BR09CA262	IGN	29/F	N.A.	217	**209**	5.45	5.24	—		V82I/E138A	C
BR09CA264	MSM	39/M	AZT/3TC/EFV	243	**629**	4.78	**<1.70**	37 months		V82I	B
BR09CA271	MSM	29/M	TDF/3TC/EFV	197	**557**	4.50	**<1.70**	26 months	M41L	M41L	B
BR09CA296	MSM	26/M	TDF/3TC/LPV/r	221	**723**	3.95	**<1.70**	29 months	K103N	K103N	B
BR09CA298	MSW	26/M	—	*731 *	668	*4.00 *	4.65	—	M230L	M230L	B
BR09CA300	MSM	21/M	—	*1062 *	729	*3.29 *	3.20	—		V82I	B
BR09SP310	MSM	27/M	TDF/3TC/RAL	108	**955**	5.55	**<1.70**	15 months		E138A	B
BR09CA343	MSM	34/M	AZT/3TC/EFV	335	388	4.41	**<1.70**	35 months		E138A	B
BR09CA344	WSM	39/F	—	*381 *	454	*3.81 *	4.42	—	K101P, K103S	K101P, K103S	B
BR09CA355	WSM	53/F	TDF/3TC/EFV	318	**787**	3.52	**<1.70**	27 months	L90M	L90M	B
BR09CA369	MSW	58/M	TDF/3TC/EFV	348	348	4.76	—	N.A		E138A	BF
BR09SP588	MSM	33/M	—	*517 *	441	*4.49 *	4.07	—	M46I, L90M/K101P, G190A	M46I, Q58E, L90M/K101P, G190A	B
BR09SP003	pARV+MSM	41/M	TDF/3TC/DRV/r/RAL	312	**490**	4.86	**<1.70**	3 months	M46I, G73S, I84V, L90M/M41L, V75M, F77L, T215D, Y188L	M46I, I54V, I84V, L90M/M41L, F77L, Y188L	B
BR09CA078	pARV+WSM	40/F	AZT/3TC/EFV	297	**492**	3.57	**<1.70**	14 months		A72V	B
BR09CA095	pARV+MSM	32/M	—	*631 *	658	*2.68 *	3.07	—	K103N, L90M	K103N, L90M	B
BR09CA187	pARV+WSM	25/F	—	*656 *	592	*3.93 *	4.24	—		V82I	B
BR09CA280	pARV	28/M	—	*632 *	395	*3.61 *	4.19	—	K103N	K103N	B
BR09CA372	pARV+MSM	29/M	TDF/3TC/ATV/r	273	**774**	*4.73 *	**<1.70**	37 months	K103N	K103N	B
BR09CA197	pARV+WSM	47/F	—	N.A.	N.A.	N.A.	N.A.	—	T215S, I85V		B
BR09SP253	pARV+WSM	47/F	—	*1142 *	1334	*2.06 *	2.70			V82I	B

^a^Samples ID (BR from Brazil, 09 for year of collection, and CA or SP for clinical site and samples number).

^b^Exposure as MSM men that refers to sex with other men, WSM for women that refers to sex with men, IDU for intravenous drug use, and pARV for patients referring to sex with one or more partners using antiretroviral therapy.

^c^Age in years, ^d^gender as M for male, and F for female.

^e^ART treatment as first regimen or no treatment.

^f^CD4+ T cells prior to treatment start (pre-ART) and ^g^last CD4 available (bold for CD4 values higher than pre-ART, when ARV was taken);

^h^Viral load prior to ART (before ART) and ^i^last VL available. (bold for undetectable viral copies/mL, <1.70 log_10_, when ARV was taken).

^j^Time since treatment initiation until last CD4/VL determination in months.

^k,l^Resistance mutations observed (Stanford CPR and 2011 IAS list).

^m^Viral subtype at *pol* region.

N.A. for data not available.
